# Minimally Invasive Plate Osteosynthesis (MIPO) Through the Posterior Approach for the Humerus: A Cadaveric Study

**DOI:** 10.7759/cureus.35578

**Published:** 2023-02-28

**Authors:** Adhish P Singh, Vijendra D Chauhan, Sanad Kumar, Deepa Singh

**Affiliations:** 1 Orthopedics, Himalayan Institute of Medical Sciences, Swami Rama Himalayan University, Dehradun, IND; 2 Anatomy, Himalayan Institute of Medical Sciences, Swami Rama Himalayan University, Dehradun, IND

**Keywords:** intramedullary nailing, open reduction and internal fixation, minimally invasive plate osteosynthesis, fracture, humerus

## Abstract

Background

Minimally invasive plate osteosynthesis (MIPO) has been effectively used in femur and tibia fractures. MIPO in the humerus is conducted by anterior (most commonly used), lateral, and posterior approaches. However, in the anterior approach, in distal humeral diaphyseal fractures, there is a lack of adequate room for screw placement in the distal fragment for good stability. In such cases, the posterior approach for MIPO may be a propitious treatment method. However, the literature on MIPO using the posterior approach for humeral diaphyseal fractures is limited. This study aimed to evaluate the feasibility of MIPO through the posterior approach and study the association of radial nerve injury with MIPO through the posterior approach for the humerus.

Methodology

This experimental study was conducted in the Department of Orthopedics, Himalayan Institute of Medical Sciences, Dehradun, Uttarakhand, India, and 20 cadaveric arms (10 right and 10 left) of 11 embalmed (formalin) cadavers were included (seven males and four females). Cadavers were placed prone on the dissection table. The posterolateral tip of the acromion and lateral epicondyle of the humerus were used as bony landmarks that were marked under C-Arm (Ziehm Imaging, Orlando, FL, USA) using K wires (Kirschner wires, Surgical Holdings, Essex, UK). Two incisions on the posterior part of the arm were made, and the radial nerve was identified at the proximal incision. After creating a submuscular tunnel, a 3.5 mm extraarticular distal humeral locking compression plate (LCP) was introduced over the posterior surface of the humerus and fixed to the humerus distally with one screw and then adjusted proximally and fixed to the humerus with another screw in the proximal window, followed by placement of couple more screws under C-Arm. After plate fixation, the dissection was completed to meticulously explore the radial nerve. The radial nerve was examined thoroughly for any injury sustained after completion of dissection, from the triangular interval to the lateral intermuscular septum where the nerve enters the anterior chamber. The position of the radial nerve with respect to plate holes was noted. The distance from the posterolateral tip of the acromion to the lateral epicondyle was measured as humeral length. The medial and lateral points where radial nerve passed over the posterior surface of the humerus were measured from the posterolateral tip of the acromion and compared with the humeral length.

Results

In this study, the radial nerve was lying over the posterior surface of the humerus for a mean distance of 52.161 ± 5.16 mm. The mean distance at which the radial nerve crossed the medial and lateral borders of the posterior surface of the humerus, measured from the posterolateral tip of the acromion, was 118.34 ± 10.86 mm (40.07% of humeral length) and 170 ± 12.30 mm (57.57% of humeral length), respectively, and the mean humeral length in this study was 295.27 ± 17.94 mm. The radial nerve and its branches were found to be intact in all cases. The radial nerve was related to the fifth, sixth, and seventh holes, with the nerve lying most commonly over the sixth hole (3.5 mm extraarticular distal humerus locking plate).

Conclusions

The posterior approach of MIPO in humeral fractures is a safe and reliable treatment modality with minimal risk of radial nerve injury. The radial nerve can be safely identified at the spiral groove using the bony landmarks described in our study.

## Introduction

Humerus diaphyseal fractures comprise 3% of all fractures and approximately 14% of all humerus fractures [1]. Humeral diaphyseal fractures show a bimodal distribution, with a first peak at 30 years and a second peak at 60-70 years of age (more in females) with no sex preponderance in younger patients [2].

There is a big armamentarium of treatment options available for the management of humeral diaphyseal fractures - conservative treatment, external fixation, intramedullary nailing, open reduction and internal fixation (ORIF) with plating, and minimally invasive plate osteosynthesis (MIPO). Nonoperative treatment has shown satisfactory results in closed fractures, but with complications like shortening and varus deformity (no specificity for the age), most surgeons opt for surgical fixation [3,4].

Although there is a mixed consensus among orthopedics on which modality is better, most surgeons prefer ORIF with plating as the treatment of choice for humeral diaphyseal fractures [4]. However, ORIF with plating is associated with complications such as periosteal stripping and soft-tissue damage, leading to delayed bone healing, iatrogenic injury to neurovascular structures, most particularly to the radial nerve (5.1% and 17%), and cosmetically bigger scar [5].

MIPO has emerged as a novel method for the surgical treatment of long bone fractures. MIPO has been effectively used in fractures of the femur and tibia [6]. The incisions are small and remote from the fracture site to avoid direct fracture exposure. MIPO provides early healing by avoiding soft tissue damage due to open reduction, preserving local vasculature [7], and avoiding callus disruption, thus achieving biological fixation [5,6]. In humerus shaft fractures, MIPO has been introduced recently and has shown to have considerable merit over traditional treatment methods, as it is not associated with any shoulder complications seen with intramedullary nailing [8], provides better postoperative functional outcome, and avoids complications of ORIF [4,5]. MIPO in the humerus is conducted by anterior, lateral, and posterior approaches, with the anterior approach being the most commonly used [5].

However, the pitfall of the anterior approach is that in distal humeral diaphyseal fractures, there is a lack of adequate room for screw placement in the distal fragment to achieve good stability. In such cases, the posterior approach for MIPO may be a propitious treatment method [5]. However, the literature available on MIPO using the posterior approach for humeral diaphyseal fractures is limited. Therefore, this study was undertaken to assess the feasibility of MIPO through a posterior approach for the humerus and evaluate the risk of radial nerve injury with the posterior approach.

## Materials and methods

This cadaveric study was conducted in the Department of Orthopedics, Himalayan Institute of Medical Sciences (HIMS), Dehradun, Uttarakhand, India.

Sample size 

A total of 20 cadaveric arms (10 right and 10 left) of 11 embalmed adult cadavers were included (seven males and four females). Cadavers with evidence of trauma (proximal one-third humerus fracture, crush injury, or mutilation) and deformity of the upper limb.

Surgical procedure

**Figure 1:**
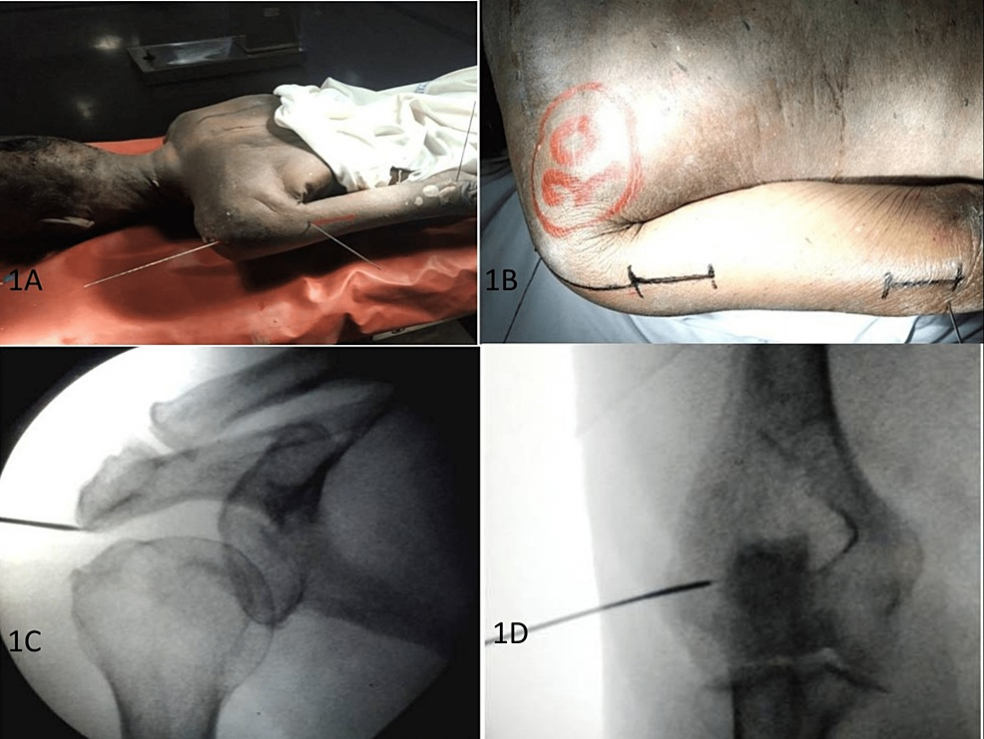
Bone land markings under C-Arm using K-wires: (A) cadaver placed in the prone position; (B) incisions marked over the posterior aspect of the arm; (C) posterolateral tip of the acromion marked using K-wires under C-Arm; and (D) lateral epicondyle marked using K-wires under C-Arm. C-Arm: an advanced medical imaging device based on X-ray technology. K-wires: Kirschner wires are stiff, straight wires that are sometimes needed to repair a fracture.

Cadavers were placed prone on the dissection table. The posterolateral tip of the acromion and the lateral epicondyle of the humerus were used as bony landmarks in this study. All the bony landmarks are marked under C-Arm (an advanced medical imaging device based on X-ray technology; Ziehm Imaging, Orlando, FL, USA) using Kirschner wires (K wires, Surgical Holdings, Essex, UK; Figure 1).

**Figure 2:**
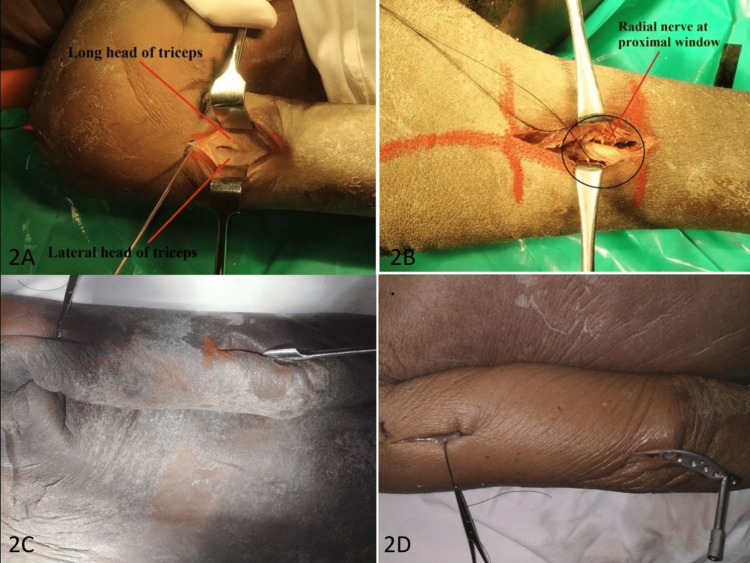
Creation of an extraperiosteal, submuscular tunnel under triceps: (A) proximal window created (between long and lateral head of triceps); (B) radial nerve explored at the proximal window; (C) submuscular tunnel created using tunneling instrument; and (D) plate introduced through the distal window.

From the posterolateral tip of the acromion, 10 cm distally, a 5 cm incision was made in the midline on the posterior aspect of the proximal arm. The surgical plane was created through the lateral head and long head of the triceps, and the radial nerve was exposed along with profunda brachii vessels (Figure 2). A 5 cm distal incision, starting proximal to the olecranon tip, was made on the posterior aspect of the distal arm over the lateral epicondyle, lateral to the olecranon fossa. The distal humerus was exposed by opening the triceps aponeurosis. Using a tunneling instrument introduced through the distal window, an extraperiosteal, submuscular tunnel was created under the triceps, protecting the radial nerve at the proximal incision site (Figure 2).

**Figure 3:**
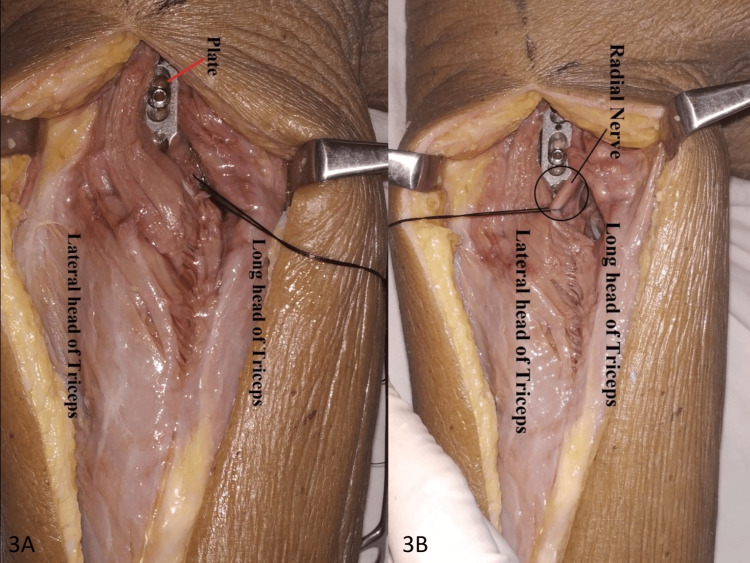
Fixation of screws to the humerus under C-Arm: (A) fixation of an extraarticular distal humeral LCP; (B) after superficial dissection (the intact radial nerve). LCP, locking compression plate

A 3.5 mm extraarticular distal humeral locking compression plate (LCP) was introduced over the posterior surface of the humerus and fixed to the humerus distally with one screw and then adjusted proximally and fixed to the humerus with another screw in the proximal window, followed by placement of couple more screws under C-Arm (Figure 3).

**Figure 4:**
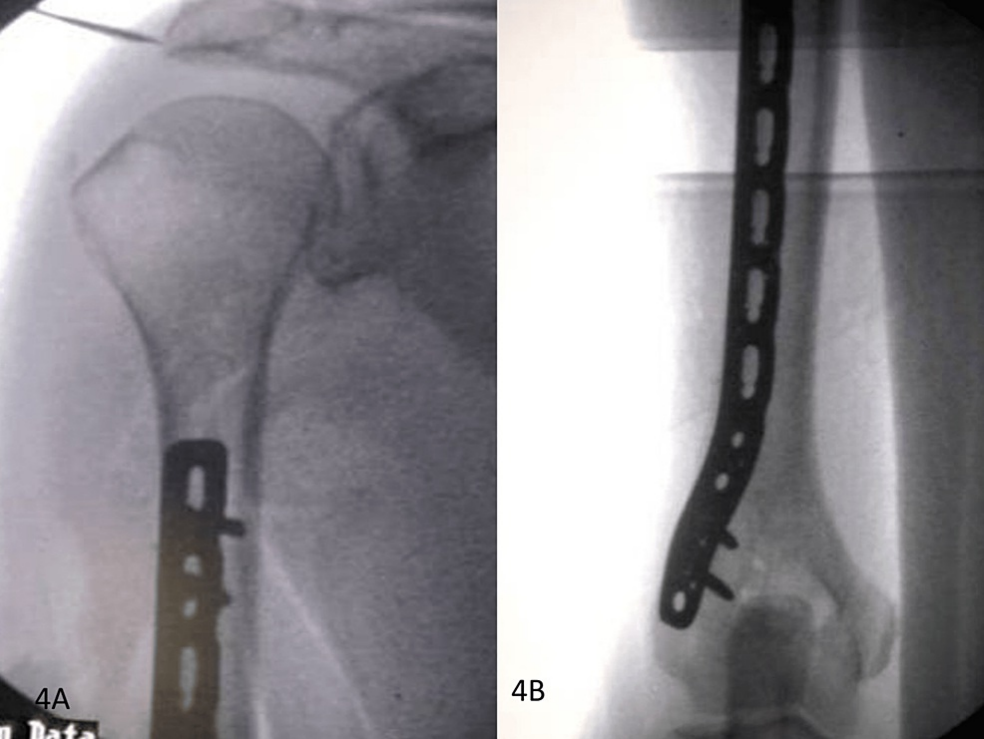
X-ray of the humerus: (A) radiographs (AP view) showing extraarticular distal humeral LCP fixed to the humerus; (B) radiographs (lateral view) showing extraarticular distal humeral LCP fixed to the humerus. AP, anterior-posterior; LCP, locking compression plate

After plate fixation, the dissection was completed to meticulously explore the radial nerve. The radial nerve was examined thoroughly for any injury sustained after completion of dissection, from the triangular interval to the lateral intermuscular septum where the nerve enters the anterior compartment. The position of the radial nerve with respect to plate holes was noted (Figure 4).

**Figure 5:**
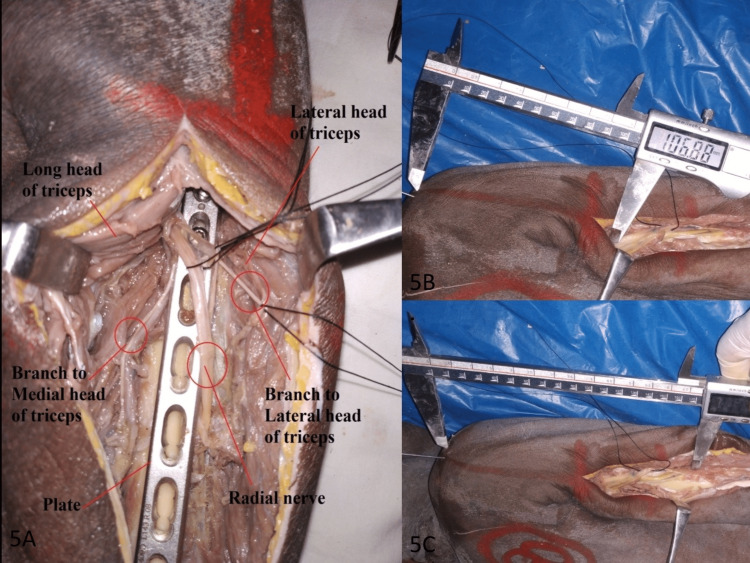
Radial nerve examination: (A) radial nerve and its branches after completion of dissection; (B) the distance (starting point) at which the radial nerve passed the medial and lateral borders of the humerus measured using a digital Vernier caliper (India Tools & Instruments Co., Mumbai, Maharashtra, India); (C) the distance (endpoint) at which the radial nerve passed the medial and lateral borders of the humerus measured using a digital Vernier caliper.

The distance from the posterolateral tip of the acromion to the lateral epicondyle was measured as the humeral length. The medial and lateral points where the radial nerve passed over the posterior surface of the humerus were measured from the posterolateral tip of the acromion and compared with the humeral length (Figure 5).

**Figure 6:**
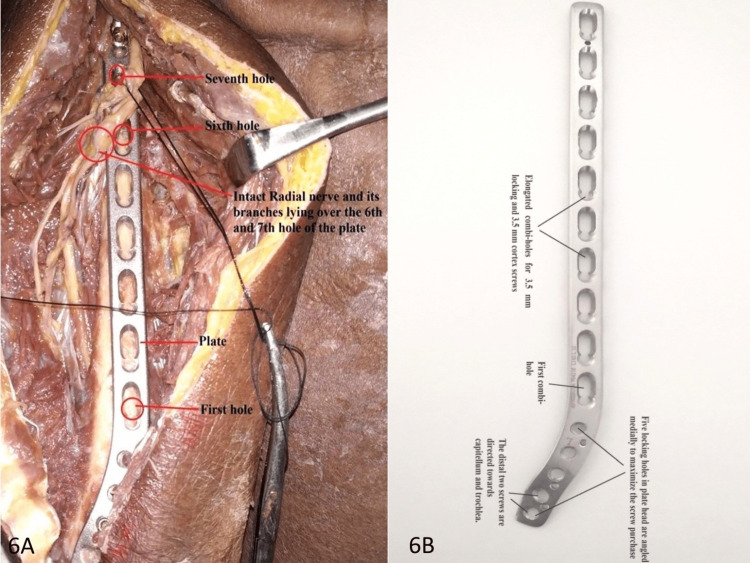
Measurement of the distance for which the radial nerve was in contact with the humerus in the spiral groove: (A) the intact radial nerve in the spiral groove lying over the sixth and seventh holes of the plate; (B) the plate used.

The distance for which the radial nerve was in contact with the humerus in the spiral groove was measured. It corresponds to the spiral groove and is the site for the proximal window where the radial nerve can be easily identified. All the measurements were taken by two independent observers for each arm using a digital Vernier caliper (India Tools & Instruments Co., Mumbai, Maharashtra, India; Figure 6), and the mean value was calculated for that measurement.

## Results

The mean humeral length of 20 cadaveric arms in our study was found to be 295.27 ± 17.94 mm, with a range of 270.52 to 312.59 mm. The mean humeral lengths were found to be 307.82 ± 4.6 mm in males and 271.96 ± 0.96 mm in females.

The mean distance at which the radial nerve passed the medial and lateral borders was 40.07% and 57.57% of the mean humeral length, respectively. The mean distance at which the radial nerve passed the medial and lateral borders in males was 40.42% and 57.84% of the mean humeral length, respectively, and in females, it was 39.34% and 56.90% of the mean humeral length, respectively.

In this study, the distance for which the radial nerve was in contact with the posterior surface of the humerus was found to be a mean value of 52.161 ± 5.16 mm for 20 cadaveric arms. The mean distance for which the radial nerve was in contact with the posterior surface of the humerus in males and females was 53.653 ± 5.15 and 49.38 ± 4.21 mm, respectively. It corresponds to the spiral groove and is the site for the proximal window where the radial nerve can be easily identified. The distribution of various variables in our study is shown in Table 1.

**Table 1:** Mean distribution of all the parameters included in the study. SD, standard deviation

Parameters	Overall, Mean ± SD (mm) (*n *= 20)	Mean ± SD (mm), males (*n *= 13)	Mean ± SD (mm), females (*n *= 7)
Humeral length	295.27 ± 17.94	307.82 ± 4.6	271.96 ± 0.96
Distance at which the nerve crossed the medial border of the humerus	118.35 ± 10.86	124.43 ± 8.05	107.04 ± 3.78
Distance at which the nerve crossed the lateral border of the humerus	170 ± 12.30	178.09 ± 5.9	154.99 ± 2.33
Radial nerve in the spiral groove	52.161 ± 5.16	53.653 ± 5.15	49.38 ± 4.21

We used a 3.5 mm extraarticular distal humerus locking plate for fixation, and the position of the radial nerve with respect to plate holes was noted. It was found that the nerve was lying most commonly on the sixth hole of the plate followed by the seventh and fifth holes, with holes counted distal to proximal from the first oblong hole of the plate lying near the olecranon fossa. No other hole was found to be related to the radial nerve. In females, the radial nerve was lying most commonly on the sixth hole of the plate followed by the fifth hole, with the nerve not related to the seventh hole in any case, while in males, the nerve was lying most commonly on the sixth hole of the plate followed by the seventh and fifth holes (Table 2). The incidence of the seventh hole was found to be more than that of the fifth hole in males.

**Table 2:** Frequency of the plate holes over which the radial nerve was lying.

Hole of the plate	Frequency (%), overall (*n *= 20)	Frequency (%), females (*n* = 7)	Frequency (%), males (*n* = 13)
Fifth	10 (50)	5 (71.4)	5 (38.5)
Sixth	19 (95)	7 (100)	12 (92.3)
Seventh	9 (45)	0 (0)	9 (69.2)

## Discussion

MIPO has evolved as a new concept for treating fractures of long bones of the upper and lower extremities. The introduction of LCPs with the concept of bridge plating has simplified the technique of MIPO by providing relative stability. MIPO has been traditionally used for treating tibia and femur fractures, but the use of MIPO in treating humerus diaphyseal fractures has gained popularity in recent years [5].

Fernández Dell'Oca first described the concept of helical implants and the use of helical plates through MIPO for the treatment of long bone fractures. He suggested using helical plates through MIPO for humerus fractures, with a curved plate positioned laterally in the proximal part and anteriorly in the distal part [9]. Perren revolutionized the concept of biological fixation and interfragmentary strain that lead to the use of bridge plating in diaphyseal fractures [10]. Livani and Belangero used a dynamic compression plate (DCP), inserted percutaneously through an anterior approach, for bridge plating of the humerus [11]. Since then, there have been innumerable studies on treating humeral diaphyseal fractures with MIPO using the anterior approach. However, fractures involving the distal third humerus, extending up to the olecranon fossa, are difficult to fix using the anterior approach for MIPO, leaving not enough bone for screw fixation to create a stable construct. In such situations, MIPO through a posterior approach is an optimal method of treatment [5].

Balam and Zahrany showed excellent union rates and functional outcomes on 37 patients with humeral diaphyseal fractures treated using a posterior approach for minimally invasive plating and suggested the use of MIPO through a posterior approach for treating middle and distal third humeral fractures [12]. Gallucci et al. showed similar results on 21 humeral diaphyseal fractures treated with MIPO through a posterior approach, with a good range of motion of both shoulder and elbow joints [5]. In a cadaveric study, Jiamton et al. also showed that MIPO through a posterior approach for the humerus is a safe method for surgically fixing middle and distal humeral diaphyseal fractures [13]. As not many studies are available on MIPO through a posterior approach, we did this cadaveric study to show the feasibility of MIPO through a posterior approach in humeral shaft fractures.

Jiamton et al. found the mean humeral length in their cadaveric study to be 268.6 mm, with a range of 238.23 to 296.61 mm, measured from the posterolateral acromion tip to the lateral epicondyle [13]. Carlan et al. and Jain et al. also measured the mean humeral length from the acromion tip to the lateral epicondyle [14,15]. The slight differences in the values of the humeral length in our study and other studies might be due to geographical variations among the cadaveric heights. In a Korean cadaveric study, Cho et al. measured the humeral length as the mean distance from the transepicondylar axis to the acromial tip and found it to be 289.5 mm [16]. The mean humeral length in their study was 302 mm in males and 278.1 mm in females. In a Turkish study, Ozden et al. measured the humeral length as the mean distance between the greater tuberosity and lateral epicondyle and found it to be 287.3 ± 19.7 mm [17].

Jiamton et al. found the mean distance of the radial nerve crossing the medial and lateral borders of the posterior humerus surface, measured from the posterolateral tip of the acromion, to be 104.7 mm (31.7%-45.6% of the humeral length; average 39%) and 142.7 mm (46.8%-62.4% of the humeral length; average 53.1%), respectively [13]. Carlan et al. measured the distance from the lateral epicondyle to the proximal and distal part of the radial nerve in the spiral groove and found it to be 17.1 ± 1.6 to 10.9 ± 1.5 cm respectively, with the radial nerve lying in the spiral groove for an average distance of 6.3 ± 1.7 cm [14]. Similarly, Jain et al., in their cadaveric study, measured the distance of the entry and exit of the radial nerve in the spiral groove from medial and lateral epicondyles, respectively, to be 18.5 ± 0.79 and 11.34 ± 0.41 cm, and stated this as the proximal safe zone of the radial nerve [15]. Gerwin et al. found that the radial nerve was 20.7 ± 1.2 cm above the medial epicondyle and 14.2 ± 0.6 cm above the lateral epicondyle and was lying on the posterior humerus surface for a distance of 6.5 cm [18].

The mean distance for which the radial nerve was in contact with the posterior surface of the humerus in our study was found to be 52.161 ± 5.16 mm for 20 cadaveric arms, 53.653 ± 5.15 mm for males, and 49.38 ± 4.21 mm for females (Table 3).

**Table 3:** Comparison of various parameters in this study with other studies. HL, humerus length; D M (mm), the distance at which the radial nerve passed the medial border of the humerus; D/H (%) M, the distance at which the radial nerve passed the medial border of the humerus (%) with respect to the humeral length; D L (mm), the distance at which the radial nerve passed the medial border of the humerus; D/H (%) L, the distance at which the radial nerve passed the medial border of the humerus (%) with respect to the humeral length; D RN SG, the mean distance of the radial nerve in the spiral groove (mm)

	Country						
	India	Thailand	South Korea	India	United States	Turkey	United States
Authors	This study	Jiamton et al. [13]	Cho et al. [16]	Jain et al. [15]	Carlan et al. [14]	Ozden et al. [17]	Gerwin et al. [18]
HL overall (mm)	295.27 ± 17.94	268.6		309.6 ± 12.3	287 ± 25	-	-
HL (mm) (Male)	307.82 ± 4.6	-	-	-	-	-	-
HL (mm) (Female)	271.96 ± 0.96	-	-	-	-	-	-
D M (mm)	118.34 ± 10.85	104.7	-	-	-	-	-
D/H (%) M	40.07	39	46.70	-	-	-	-
D L (mm)	170 ± 12.30	142.7	-	-	-	-	-
D/H (%) L	57.57	53.10	60.50	-	-	-	-
D RN SG (mm) (Overall)	52.161 ± 5.16	-	-	43 ± 0.75	63 ± 1.7	58.4 ± 10.3	65
D RN SG (mm) (Male)	53.653 ± 5.15	-	-	-	-	-	-
D RN SG (mm) (Female)	49.38 ± 4.21	-	-	-	-	-	-

We used a 3.5 mm extraarticular distal humerus LCP for fixation and assessed the position of the radial nerve with respect to the plate holes and found that the nerve was lying most commonly on the sixth hole of the plate followed by the seventh and fifth holes. No other hole was found to be related to the radial nerve. The importance of these holes with respect to radial nerve injury has already been stressed in our results. We also suggest the use of a longer plate with 10 holes, especially in males, for the posterior approach of MIPO in the humerus to allow enough screws in the proximal segment for good stability without injuring the radial nerve during screw placement.

In this study, the radial nerve was found to be intact in all 20 cases of plating through the posterior approach of MIPO in the humerus. Jiamton et al. had similar results in their cadaveric study with no radial nerve injury seen [13]. Gallucci et al. and Balam and Zahrany also suggested that MIPO through a posterior approach in humeral fractures was safe when considering radial nerve injury [5,12].

Therefore, we find MIPO through the posterior approach for the humerus a safe procedure and recommend its use as a treatment modality for treating humeral shaft fractures. Proper technique of tunneling for preparation of submuscular tunnel and surgical dissection at the proximal window play a crucial role in protecting the radial nerve. To prevent injury to the radial nerve during humerus plating, open or through minimally invasive technique, one must be well versed with the local anatomy, the relationship of the radial nerve with the posterior surface of the humerus and the spiral groove, and the triceps muscle.

Limitations of this study

There are a few limitations of this study. We used an intact humerus instead of a fractured humerus model on which it is easier to perform the procedure. In a fractured humerus, the correct assessment of the anatomy can only be made after fracture reduction. Also, soft-tissue damage will alter the local anatomy. Also, it is easier to manipulate the nerve in a cadaver with no risk of nerve injury than in a live patient during surgery where nerve handling is done very cautiously.

## Conclusions

After evaluating the results of this experimental study, we conclude that the posterior approach for MIPO in humeral fractures is a safe and reliable treatment modality with minimal risk of radial nerve injury. Meticulous dissection is essential for the preservation of the radial nerve and its branches. The radial nerve can be safely identified at the spiral groove using the bony landmarks described in our study. In this study, the radial nerve was in contact with the posterior humerus surface for a mean distance of 52.161 ± 5.16 mm, and the mean distance at which the radial nerve passed the medial and lateral borders of the posterior humerus surface when measured from the posterolateral acromion tip was 118.34 ± 10.86 mm (40.07% of the humeral length) and 170 ± 12.30 mm (57.57% of the humeral length), respectively. Also, the mean humeral length in our study was 295.27 ± 17.94 mm. Hence, we can conclude that in females, the fifth and sixth holes are of great importance, while in males, all three holes, the fifth, sixth, and seventh holes, are important when operating humeral diaphyseal fractures with MIPO through a posterior approach using a 3.5 mm extraarticular distal humerus LCP, to avoid injuring the radial nerve during screw placement. These values can be reflected in the Indian population to identify the location of the radial nerve over the posterior surface of the humerus. We suggest the use of longer plates with 10 holes to avoid radial nerve injury in the proximal window during screw placement, considering the relationship of the radial nerve with the fifth, sixth, and seventh holes, and allow placement of enough screws in the proximal segment for good stability without injuring the nerve.
